# Multiple dsRNases Involved in Exogenous dsRNA Degradation of Fall Armyworm *Spodoptera frugiperda*

**DOI:** 10.3389/fphys.2022.850022

**Published:** 2022-05-05

**Authors:** Yang Yao, Dong-Jiang Lin, Xiang-Yun Cai, Ran Wang, You-Ming Hou, Chao-Hua Hu, San-Ji Gao, Jin-Da Wang

**Affiliations:** ^1^ National Engineering Research Center of Sugarcane, Fujian Agricultural and Forestry University, Fuzhou, China; ^2^ State Key Laboratory of Ecological Pest Control for Fujian and Taiwan Crops, Ministry of Education, College of Plant Protection, Fujian Agricultural and Forestry University, Fuzhou, China; ^3^ Institute of Plant Protection, Beijing Academy of Agriculture and Forestry, Beijing, China

**Keywords:** RNA inference, dsRNase, efficiency, liposome, *Spodoptera frugiperda*

## Abstract

RNAi is regarded as a promising technology for pest control. However, not all insects are sensitive to RNAi. Studies have confirmed that insect dsRNases are one of key factors affecting RNAi efficiency. In the current study, we identified four genes coding for dsRNases from the *Spodoptera frugiperda* genome. Spatial and temporal expression analysis showed that those dsRNases were highly expressed in the midgut and old larvae. Then a delivery method was applied for inducing efficient RNAi based on dsRNA encapsulated by liposome. Furthermore, we assessed degradation efficiency by incubation with dsRNA with gut juice or hemocoel to characterize potential roles of different SfdsRNases after suppression of *SfdsRNase*. The result showed that interferenced with any *sfdsRNase* reduced the degradation of exogenous dsRNA in midgut, interfered with *sfdsRNase1* and *sfdsRNase3* slowed down the degradation of exogenous dsRNA in hemolymph. Our data suggest the evolutionary expansion and multiple high activity dsRNase genes would take part in the RNAi obstinate in *S. frugiperda*, besides we also provide an efficient RNAi method for better use of RNAi in *S. frugiperda*.

## Introduction

RNA interference (RNAi) is a conserved regulatory process to induce sequence-specific gene silencing effect and modulated gene expression in a controlled manner. Therefore, the idea of silencing specific fatal genes in insects has been suggested as a potential strategy for pest management, such as expression of dsRNA in transgenic plants or by spraying absorbable dsRNA pesticide (Baum et al., 2007; Kunte et al., 2020; Zhu and Palli, 2020). Interestingly, the successful systemic RNAi has been established as an important tool for the development of potential insecticidal dsRNAs that can target essential genes in many other tissues of the insect pests (Huvenne and Smagghe, 2010). More recently, the use of the sprayable double-strand RNA-based biopesticide technology was proven to be effective strategy for controlling Colorado potato beetle *Leptinotarsa decemlineata* by GreenLight Biosciences (Rodrigues et al., 2021). Not all insects, viz., Lepidopteran and Hemipteran orders, however, show sensitivity to RNAi (Terenius et al., 2011) compared to the Coleopteran order (Wang et al., 2013; Guo et al., 2021). Furthermore, to induce systemic RNAi in these highly susceptible insects, a particularly intriguing aspect of RNAi is that the dsRNA is not only capable of entering gut cells but can spread to other tissues as well (Joga et al., 2016). Several factors have been attributed to induce the variability of insect RNAi responses, such as the malfunction or deficiency of core RNAi machinery components, difficulty of cellular dsRNA uptake and systemic distribution, and fast digestion of dsRNA by double-stranded ribonucleases (dsRNases). The existence of dsRNases is deemed as one of the major obstacles influencing successful RNAi by affecting dsRNA integrity in body fluids (Shukla et al., 2016; Wang et al., 2016).

The dsRNases were first isolated from silkworm *Bombyx mori* (Arimatsu et al., 2007; Yuji et al., 2007). Subsequently, large amounts of dsRNases were identified from various insects (Prentice et al., 2019; Peng et al., 2020). However, the function of them was remained to be characterized. Generally, the dsRNases were participating in dsRNA degrading and reduced RNAi efficiency (Yuji et al., 2007; Song et al., 2019). For instance, five dsRNases were identified from the *S. litura,* and functional analysis revealed they may contribute to the lower and variable RNAi efficiency (Peng et al., 2020). While the report from cotton bollworm (*Helicoverpa armigera*) demonstrated an entirely different result: RNAi efficiency was not significantly upregulated when knocking out *HaREase* by CRISPR/Cas9 system (Guan et al., 2019). Therefore, a study of molecular and functional characteristics of dsRNases would make contributions for better applications of RNAi in both scientific research and pest management area.

The fall armyworm *Spodoptera frugiperda* is a devastating agricultural pest in many countries, the outbreak of this pest often leads to significant loss of many commercial crops such as corn, rice, and sugarcane. Repeated use of chemical pesticides causes a considerable decrease in pesticide resistance (Chi et al., 2021; Hafeez et al., 2021; Paredes-Sanchez et al., 2021). Therefore, the demand for new controlling strategies is eagerly excepted. The use of RNAi would provide a new way for *S. frugiperda* management. However, limited successful RNAi cases were reported (Christiaens et al., 2020; Kunte et al., 2020; Zhu and Palli, 2020). Injection of dsSfV-ATPase resulted in enlarged midgut and difficulty of nutrient absorption, significant lethal effect was only observed by continuous dsRNA feeding (Parsons et al., 2018). However, it is still unknown the details of dsRNases in *S. frugiperda* and whether those dsRNases contributed to lower RNAi efficiency. With the help of up-to-date genome sequencing technology, it is possible to identify functional genes from genome-scale. Hence, in the current study, we identified genes coding for dsRNases from the published *S. frugiperda* genome database (http://v2.insect-genome.com/Organism/715); then a delivery method was applied for inducing efficient RNAi *in vivo* based on previous studies in Sf9 cell lines (Gurusamy et al., 2020b); in addition, an assay was performed by suppressed those identified *SfdsRNases via* our efficient delivery method and assessed digestion efficiency by incubation with dsRNA with gut juice or hemocoel to characterize potential roles of different *SfdsRNases* on RNAi efficacy in *S. frugiperda*.

## Materials and Methods

### Insect Rearing

The insects were originally collected from the countryside of Fuzhou, Fujian Province, China, and reared in a laboratory climate chamber at 26°C, relative humidity of 66%, and light for 14 h/10 h. Newly hatched larvae were fed with an artificial diet until pupation, the diet containing soybean powder, wheat bran, yeast powder, casein, ascorbic acid, choline chloride, sorbic acid, inositol, streptomycin, penicillin sodium, propylparaben and agar (Li et al., 2019). Then the adult was supplied with 10% honey solution for oviposition on fresh corn leaves.

### Identification of *dsRNase* Gene Family of *S. frugiperda*

The genomic data of *S. frugiperda* was downloaded from the InsectBase2.0 (http://v2.insect-genome.com/) website. The conserved domain of Endonuclease_NS (PF01223) was downloaded from the protein family database (Pfam, http://pfam.xfam.org/) to search for the *dsRNase* gene family. The *dsRNase* genes were retrieved from the *S. frugiperda* genome database by using HMMER software under Linux system (Potter et al., 2018) with default parameters and significant e-value of 0.01. To ensure the complete identification of the *SfdsRNase* gene family, redundant and duplicated sequences were manually removed by confirmation using the BLASTX algorithm against a non-redundant protein database (https://blast.ncbi.nlm.nih.gov/Blast.cgi).

### Bioinformatic Analysis of dsRNase From *S. frugiperda*

The online website Gene Structure Display Server version 2.0 (GSDS, http://gsds.cbi.pku.edu.cn/) was used to conduct gene structure analysis. The amino acid motifs present in the predicted SfdsRNase protein sequences were analyzed using the MEME program (http://meme-suite.org/tools/meme). The motif distribution type was set as 0 or 1 occurrence per sequence with the default setting. TBtools (Toolbox for biologists) (Chen et al., 2020) was then used for visual analysis.

The position data for the *SfdsRNase* gene family on the chromosomes was obtained from the genome annotation file, and the chromosomal locations of the *SfdsRNase* genes were visualized.

SfdsRNase protein sequences were retrieved from the NCBI database, and sequences from insects were used to conduct phylogenetic analysis (Supplementary Table S1). A maximum likelihood method was conducted in MEGA 7.0.14 (Kumar et al., 2016). Branch confidence was estimated *via* bootstrap analysis with 1,000 replicates using the default parameters, and the tree was visualized with Evolview (https://www.evolgenius.info//evolview/#login).

### RNA Extraction and RT-qPCR Analysis

To study the spatial and temporal expression, RNA from different tissue parts (head, hemolymph, midgut, epidermis dissected from ten fifth instar larvae) and four to five larvae from different instars was extracted using the Trizol reagent following the MagZol^TM^ Reagent instructions (Magen, Shanghai, China) with three biological replicates. The extracted RNA was tested for concentration with a multifunctional microplate reader and 1% agarose gel electrophoresis was used to verify the integrity and quality of the RNA. After obtaining the total RNA of *S. frugiperda*, HiScript^®^ Ⅱ Q RT SuperMix for qPCR (+gDNA wiper) reagent Kit (Perfect Real-time) (Vazyme, Nanjing, China) was used to synthesize cDNA. Total RNA and cDNA are stored at −80°C and −20°C, respectively for subsequent analysis.

Primer 5.0 software was used to design gene specific primers for cloning the detected *SfdsRNase* and *SfV-ATPase* sequences (Supplementary Table S2). Premix Taq™ (TaKaRa Taq™ Version 2.0) was used for amplification, and then amplified and purified products were connected to pMD 19-T Vector (TaKaRa, Dalian, China) for sequence verification.

Based on the cloning sequence of *SfdsRNase*, Primer 5.0 was used to design quantitative primers for nuclease genes (Supplementary Table S2). The real-time quantitative PCR was performed with ChamQ Universal SYBR qPCR Master Mix reagents (Vazyme) in an Applied Biosystems Q3 system. System configuration and program settings were performed according to the reagent instructions. β-actin was used as an internal reference gene (Rodrígues et al., 2010). Each experimental unit contains four biological replicates and three technical replicates. The 2^^−ΔΔCT^ was used to calculate the relative expression level.

### DsRNA and Nano-dsRNA Synthesis

Using the plasmids containing the *SfdsRNase* and *SfV-ATPase* genes as templates, the fragments were amplified using T7 polymerase-specific primers (Supplementary Table S2), detected by 1.5% agarose gel electrophoresis followed by purification and recovery of the fragment. To eliminate the influence of the gene background level, the exogenous gene EGFP was used as a template to synthesize dsEGFP as a negative control. Using the above-amplified product as a template, the T7 RiboMAX™ Express RNAi System (Promega, Madison, United States) was used to synthesize dsRNA separately and the dsRNA needed in the experiment was synthesized *in vitro* according to manufacturer instructions. Take out 1 μl of synthetic dsRNA, dilute it with water ten times, draw apart and use a microplate reader to measure the concentration, then take the remaining diluted solution for gel electrophoresis to test the quality of dsRNA, and finally put it in a −80°C ultra-low temperature refrigerator for later storage.

Use 0.1 M sodium acetate solution to dissolve 75% acetylated chitosan and prepare a 0.02% (w/v) chitosan (Sigma-Aldrich, Shanghai, China) solution. Add 32 μg of dsRNA to 200 μl of chitosan-sodium sulfate solution, the ratio of chitosan solution to sodium sulfate is 1:1. Incubate at 55°C for 1 min, vortex, and mix at high speed for 30 s to form a chitosan dsRNA solution.

The microwave method was used to synthesize CQD nanoparticles (Das et al., 2015). Mix 9 ml polyethylene glycol (PEG200) (TCI, Shanghai, China) with 3 ml nuclease-free water. Mix 0.4 ml of polyethyleneimine (PEI) (TCI, Shanghai, China) with 1.6 ml of nuclease-free water, and then mix with the prepared polyethylene glycol solution. Then, put it in a microwave oven and heat for 3 min, then mix the configured CQD and the sodium sulfate solution containing dsRNA at a ratio of 20:1, and finally put it in 4°C and incubate overnight.

For LIP-dsRNA complexes, dsRNA was mixed with an appropriate amount of liposome transfection reagent Lipofectamine 2000 (Thermo Fisher Science, Waltham, United States) and incubated according to the instructions.

### Administration of dsRNA by Oral Uptake and Injection

To test the RNAi efficiency of nanomaterial treated dsRNA on *S. frugiperda* larvae, we first evaluated the lethal effect of nanomaterial. Fresh 30 ml of dsEGFP with nanomaterial was added to artificial diet every day and fed with first instar for 1 week. The mortality is calculated every day. Then nanomaterial-treated dsRNA targets for *SfV-ATPase* were applied to determine RNAi efficiency by feeding method as described above. The mortality and weight were documented after 7 days of treatment. The dsEGFP diluted with ddH_2_O was used as a control. Fifty larvae were treated as a biological replicate, and the experiment was repeated three times.

Furthermore, to test the potential mechanism of nanomaterial treated dsRNA on enhancing RNAi efficiency, we used the injection method to achieve a more efficient RNAi effect. Briefly, dsEGFP were incubated LIP-dsRNA (600 ng/μl) according to the instructions of LIP 2000. Use a microsyringe to inject 4 μl LIP-dsRNA into the abdominal cavity of the fourth instar larvae. The larvae were injected with an equal volume of water-dsEGFP as control. Twenty to thirty fourth instar larvae were injected in each treatment. The experiment was repeated three times, and the treated larvae were kept fed and observed with an artificial diet. The midgut juice and hemolymph were further collected for the detection content of dsEGFP.

### Midgut Juice and Hemolymph Extraction

Midgut juice extraction refers to the extraction method (Ayra-Pardo et al., 2007). First, prepare 30–40 fourth-instar larvae and place them on ice to make them lose their activity. Then, use a dissecting needle to fix the worm body on the plate, use a scalpel to cut the abdomen, cut the midgut tissue, and place it in a PE tube containing 100 ul PBS. Put the midgut tissue at 4°C, 16000 × g, 20 min, take the supernatant into a new tube, repeat the above steps, and finally the supernatant and midgut juice, store it at −80°C.

The extraction of hemolymph refers to the method reported (Yang and Han, 2014). First, prepare 30–40 fourth instar larvae and place them on ice to make them lose their mobility. Then, use a dissecting needle to fix the worm body on the plate, use scissors to cut a small mouth in the abdomen of the worm body, and then use a 10 μl pipette tip to aspirate the hemolymph and transfer it to a nuclease-free 1.5 ml PE tube. Place the hemolymph melanin, put an appropriate amount of phenylthiourea in the PE tube in advance. The extracted hemolymph was 4°C, 16000 × g, 5 min, the supernatant was discarded, and the hemolymph extract was stored at −80°C.

### DsRNA Content Detection

To accurately monitor the dsRNA content in both *in vivo* and *in vitro* conditions. EGFP was used to eliminate the influence of gene background level, and a dsRNA with a fragment length of 414 bp was designed. After the dsRNA feeding or incubation experiment, RNeasy Micro Kit was used to extract dsRNA in each sample, and cDNA was synthesized according to HiScript^®^Ⅱ Q RT SuperMix for qPCR (+gDNA wiper) reagent Kit (Perfect Real time) (Vazyme) instructions.

Used modified qPCR method described by (Wang et al., 2020) to detect the absolute content of dsRNA in the sample. The dsRNA (10^5^,10^4^,10^3^,10^2^,10^1^,10^0^,10^−1^ pg/μl) was serially diluted and reverse transcribed into cDNA, and the CT values corresponding to different concentrations of dsRNA were detected by SYBR Green qPCR (Vazyme), and a linear regression model was established. Taking the calculated CT value as the X-axis and the Y-axis as the dsEGFP content in the sample, a linear regression model equation was constructed (Supplementary Figure.S1). After RNA extraction and RT-qPCR analysis of the sample, the corresponding CT value is substituted into the linear regression model to obtain the content of dsRNA in the sample.

### *Ex Vivo* Degradation of dsRNA in Hemolymph and Midgut Fluid

To evaluate the roles of sfdsRNases on dsRNA stability, 4 μl dsRNA (1,500 ng/μl) target to four *sfdsRNase* (sfdsRNase1–4), respectively with LIP treated were injected into fourth instar larvae as described before. Twenty-four hours later, the gene expression level of *sfdsRNase1*–*4* was detected. The hemolymph and midgut fluid were then dissected and stored from treated larvae, also as described before. *Ex vivo* assays were conducted to evaluate the capability of those fluids in dsRNA degrading: first, the dsEGFP was incubated with 200 μl hemolymph or midgut fluid extract at room temperature for 10 min and 60 min. After each time point, the reaction was stored at −20°C to stop the enzymatic reaction. Then the dsEGFP content in the samples was calculated after RNA extraction, cDNA synthesis, and qPCR detection as described above. The relative content of dsEGFP to assess the ability of midgut juice and hemolymph to degrade dsRNA. In order to more accurately record the changes of dsRNA content during in *ex vivo*, the relative content was used for calculation in the experiment. Calculate the relative content with the following formula:

$$\text{Relative content of dsEGFP}=\frac{\text{Treatment group dsEGFP content}}{\text{Control group dsEGFP content}}$$

### Statistical Analysis

IBM SPSS statistics was used to test the significance. To compare the treatment means 0.05 probability level was used. All statistical analysis is done on Prism 9.0 software (GraphPad, La Jolla, CA, United States).

## Results

### Bioinformatics Screening of *S. frugiperda* Nuclease Gene

Four *dsRNases* were obtained from *S. frugiperda* genome after deleting redundant sequences and pseudogene sequences, named as *sfdsRNase1*, *sfdsRNase2*, *sfdsRNase3,* and *sfdsRNase4* according to those in *S. litura*. The predicted full length of CDS ranges from 957 to 1,341, encoding 318 (SfdsRNase1)-446 (SfdsRNase4) amino acids. The predicted protein weight ranges from 36.01 to 50.18 kDa, and the isoelectric point ranges from 5.25 to 9.45. Except for SfdsRNase2, the remaining three amino acid sequences are predicted to contain a signal peptide. The four sfdsRNases amino acid sequences all contain the Endonuclease_NS Structure domain (Table 1; Supplementary Figure S2).

**TABLE 1:** Basic information and physical and chemical properties of four predicted *S. frugiperda* dsRNase.

Gene name	Gene ID	GenBank accession	Protein length	Mw (kDa)	Theoretical pI	Signal peptide	Endonuclease_NS (position)
*SfdsRNase1*	Sfru170710.1	OL960003	318	36.01	9.45	Yes	144–318
*SfdsRNase2*	Sfru175350.1	OL960002	395	44.80	5.66	No	135–378
*SfdsRNase3*	Sfru068710.1	OL960004	445	49.27	6.17	Yes	187–428
*SfdsRNase4*	Sfru003330.1	OM001111	446	50.18	5.25	Yes	185–429

**FIGURE 1:**
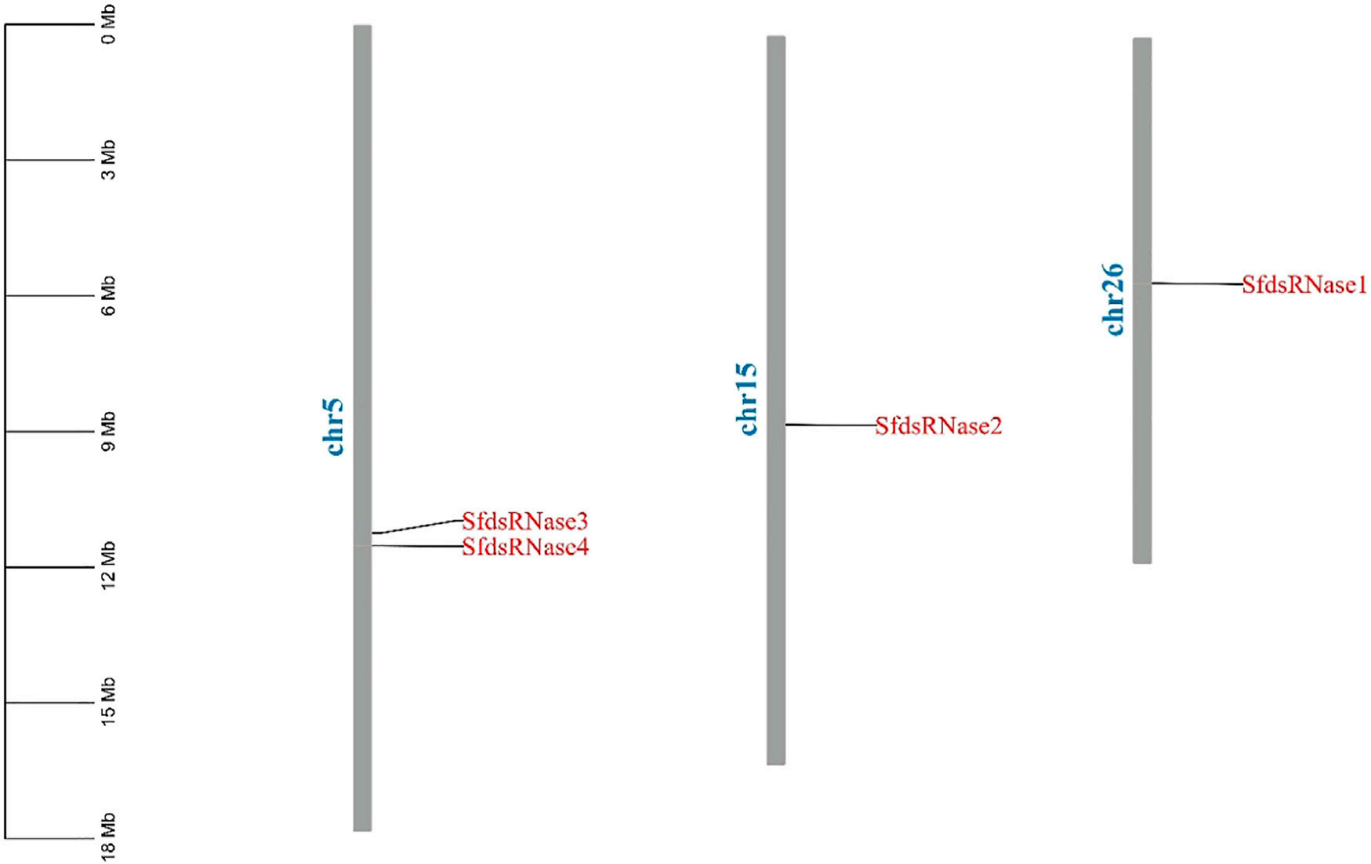
Chromosome location analysis of dsRNase gene of *S. frugiperda.* Tbtools was used to analyze the chromosome location of the *S. frugiperda* dsRNases gene.

**FIGURE 2:**
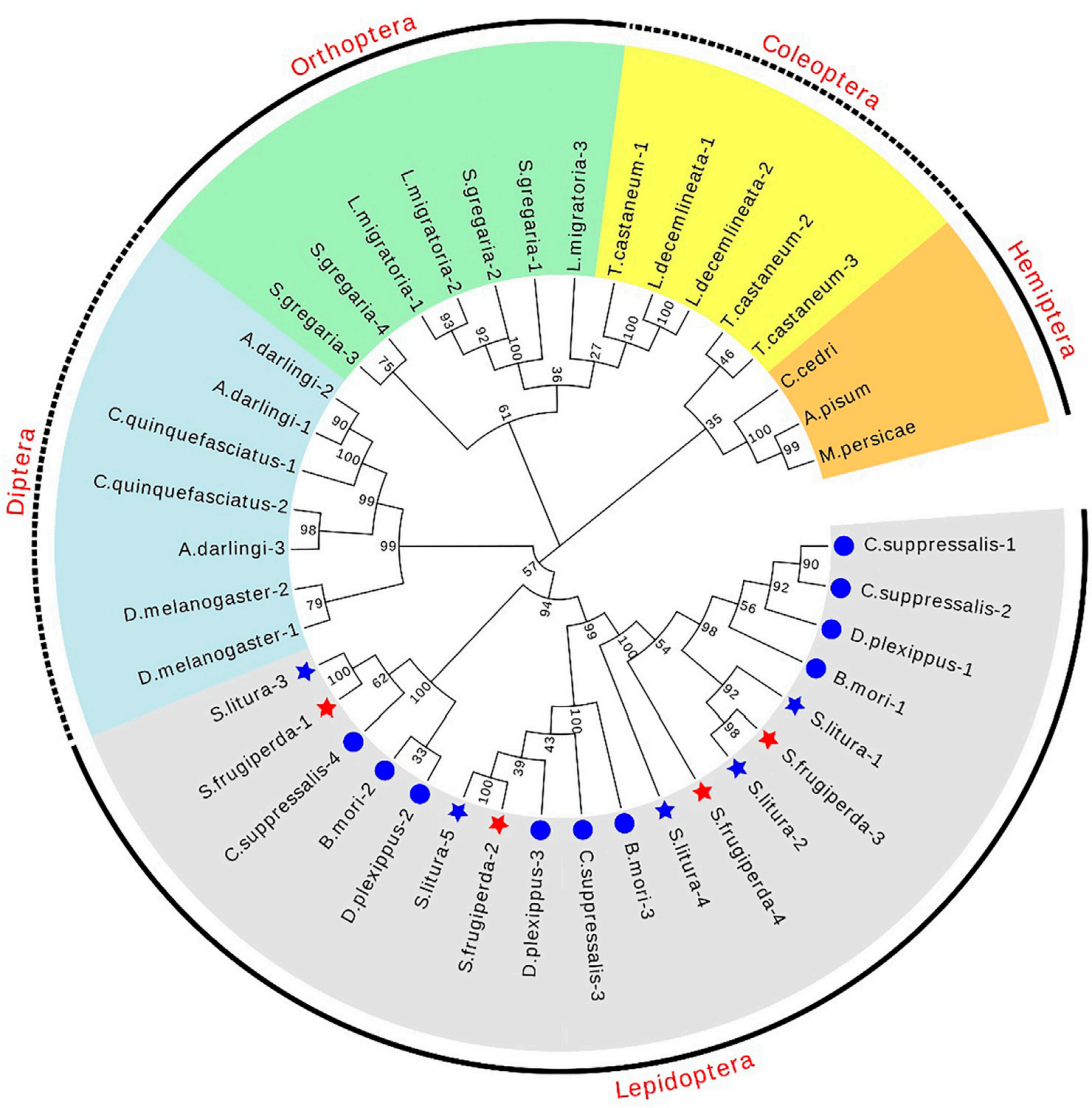
Phylogenetic tree of different species dsRNase in insects. The maximum likelihood method was used to construct phylogenetic trees of different species of dsRNase in insects. *S. frugiperda* is represented by a red star, and *S. litura* is represented by a blue star. Its four sldsRNases GenBank numbers of *S. frugiperda* dsRNase (OL960003, OL960002, OL960004, and OM001111) and *S. litura* dsRNaseare respectively (QJD55609.1), (QJD55610.1), (QJD55611.1), (QJD55612.1).

The chromosome location information shows that the four dsRNase genes are located on three chromosomes (Figure 1). In addition, a total of 41 dsRNase gene protein sequences of different species (Supplementary Table S1) were obtained from NCBI. The results of the phylogenetic analysis showed that the dsRNase genes of insects are mainly distributed in the five categories of Lepidoptera, Diptera, Orthoptera, Coleoptera, and Hemiptera orders (Figure 2). Among them, the four dsRNase genes from *S. frugiperda* are clustered to those reported in *S. litura*, indicating that the dsRNase genes of the two species are closely related.

### Spatial and Temporal Expression of Four *sfdsRNases*

**FIGURE 3:**
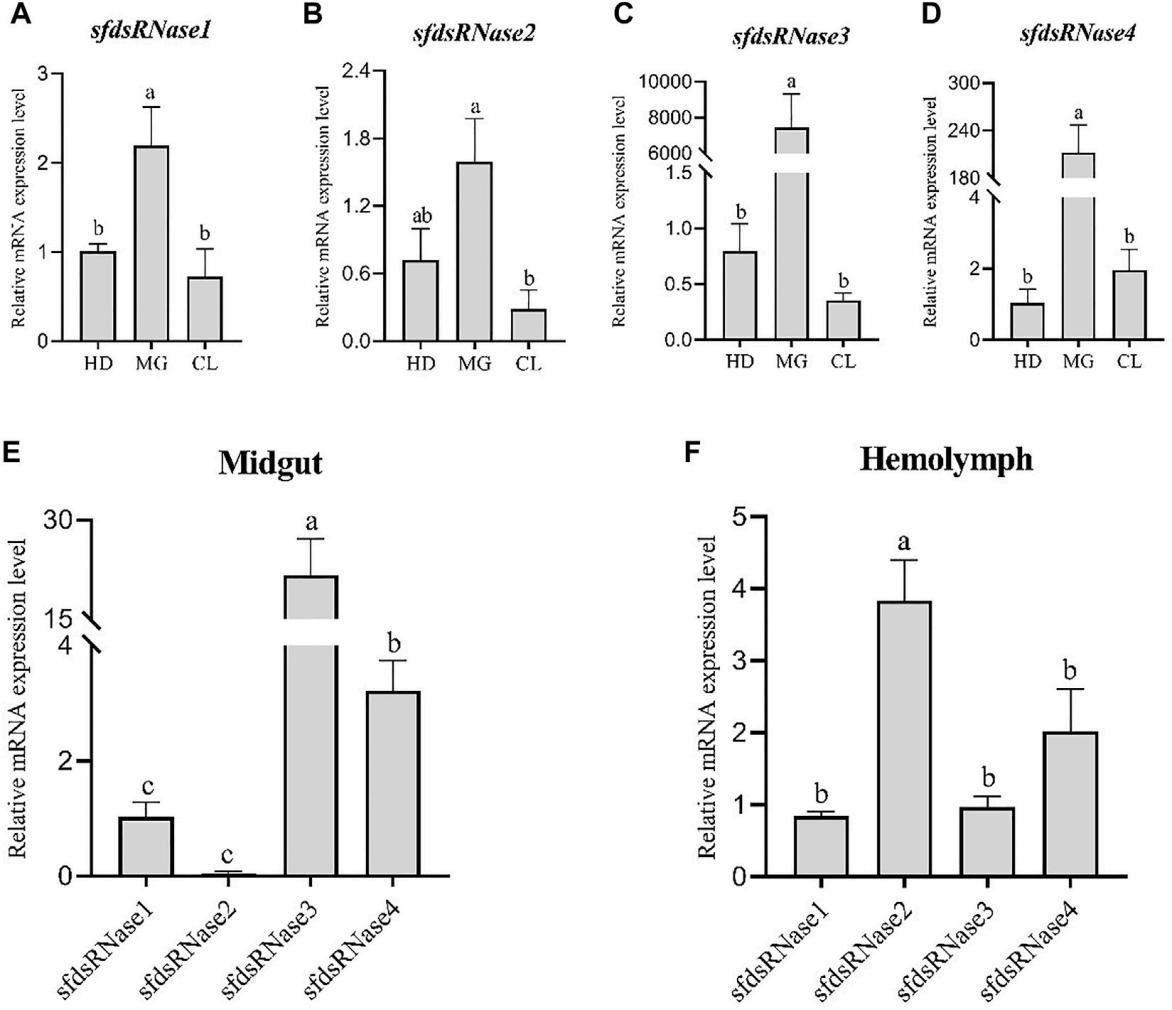
Expression analysis of *S. frugiperda* dsRNase in different tissues. Collect three different tissues of sixth instar larvae, head (HD), midgut (MG), and epidermis (CL) for RT-qPCR, and calculate the expression levels of sfdsRNases in different tissues **(A–D)**. Using *sfdsRNase1* as a control, analyze the relative expression differences of the four sfdsRNases in the midgut and hemolymph **(E,F)**. Five larvae are a biological replicate, and this experiment was performed three times in total. The data shown are mean ± SE, *n* = 15, different letters indicate a significant difference among tissues [*p* < 0.05, one-way ANOVA followed by Duncan’s multiple range test for **(A,B)**].

The results showed that sfdsRNase1–4 were all highly expressed in the midgut (Figures 3A–D), among them, the high expression of *dsRNase3* is extraordinary, and the expression level is nearly 7,000 times higher than that of the other two tissues (Figure 3C). In addition, we also compared the expression level of four *sfdsRNases* from midgut and hemolymph. The *sfdsRNase3* was most abundant in the midgut, the expression level of *sfdsRNase3* is still 400 times that of *sfdsRNase1* and more than seven times that of *sfdsRNase1*. Since the mRNA levels of the four *sfdsRNases* in hemolymph reached the detection limit, we compared them horizontally and found that *sfdsRNase2* was the highest expression level among the four *S. frugiperda* dsRNases (Figure 3F). These results indicate that the expression levels of different dsRNase in the same tissue are also quite different.

**FIGURE 4:**
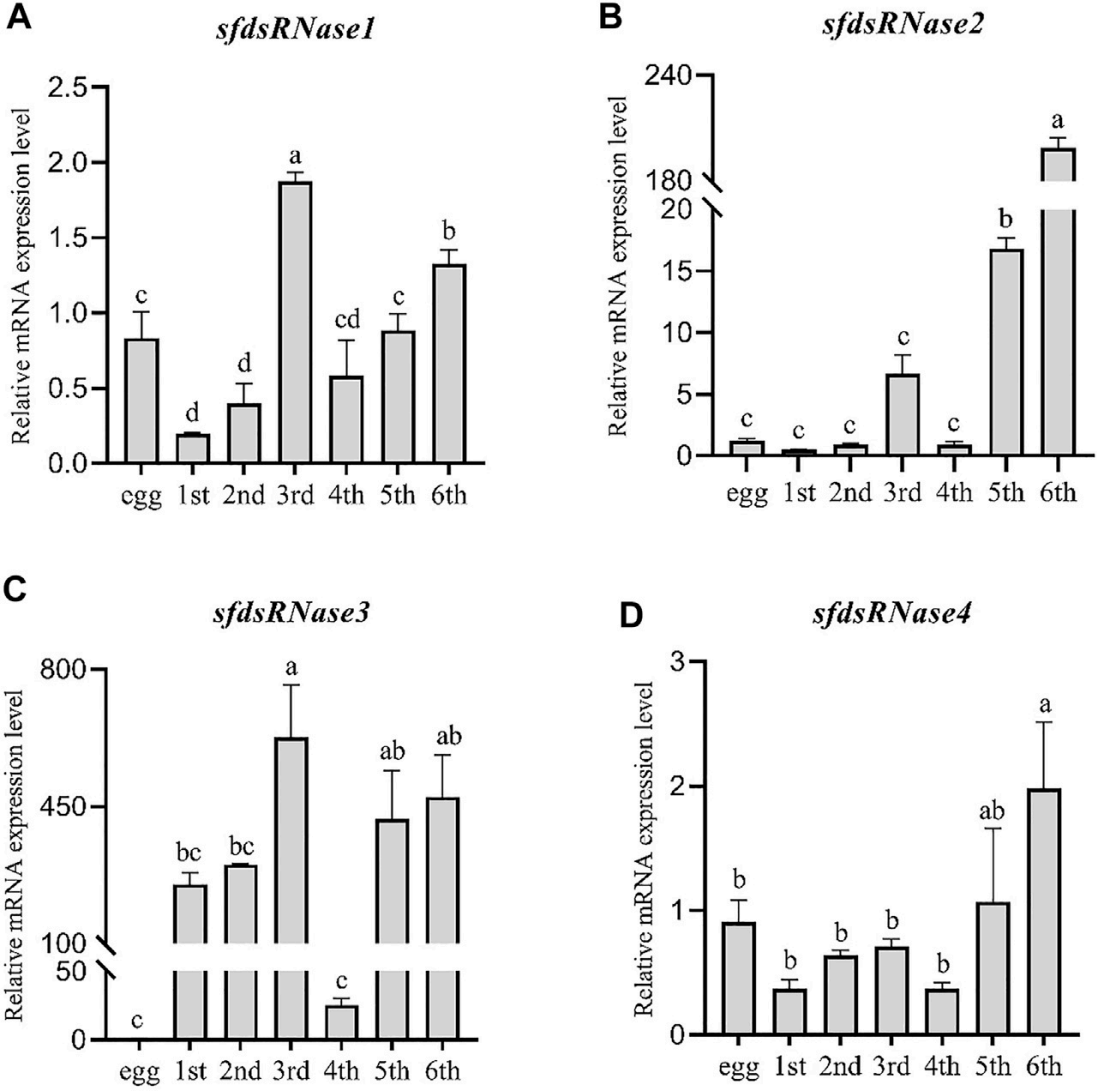
Expression analysis of *S. frugiperda dsRNases* at different ages. Using egg, first instar larvae (first), second instar larvae (second), third instar larvae (third), fourth instar larvae (fourth), fifth instar larvae (fifth), and sixth instar larvae (sixth) as templates for qPCR and using the eggs as a control to calculate the dsRNases in different instars. The relative expression level of mRNA **(A–D)**. Five larvae are a biological replicate, and this experiment was performed three times in total. The data shown are mean ± SE, *n* = 21, different letters indicate a significant difference among ages [*p* < 0.05, one-way ANOVA followed by Duncan’s multiple range test for **(A,B,C)]**.

The temporal expression levels were analyzed from the six instar stages compared to in egg. It was found that *sfdsRNase1–4* could be detected at all instars, *sfdsRNase1* and *sfdsRNase3* had the highest expression in the third instar stage (Figure 4A), *sfdsRNase2* and *sfdsRNase4* had higher expression in the fifth and sixth instar (Figures 4B,D). In addition, the mRNA expression levels of adjacent ages are also very different. The expression level of *sfdsRNase2* at the sixth age is ten times as much as that at the fifth age (Figure 4B). And the mRNA expression level of fourth instar larvae of *sfdsRNA3* was significantly lower than that of other instar larvae, only one-tenth of that in first instar larvae (Figure 4C).

### Lip-Encapsulated dsRNA Improves the Efficiency of RNAi

First, to verify whether the nanomaterial itself is toxic to *S. frugiperda* larvae, three nanomaterials were capsulated with dsEGFP and added into an artificial diet continuously for 5 days, and the same amount of dsRNA was incubated with water instead of nanomaterials as a control. We found the mortality of larvae fed with CQD-dsEGFP was significantly higher than that caused by LIP and CHS, and the mortality caused by LIP and CHS was close to the normal mortality of larvae (Supplementary Figure S3).

Due to the lethal effect of CQD, the remained LIP and CHS nanomaterials were used for further experiments. Then we synthesized dsRNA from the reported lethal target gene *SfV-ATPase*, and capsulated it with LIP and CHS. The results showed that the LIP-dsV-ATPase complex can significantly increase the mortality of *S. frugiperda* larvae, and the mortality of the larvae fed with the CHS-dsV-ATPase complex feed has no difference compared with the control. Analyzing the growth of larva, it is found that LIP-dsV-ATPase also significantly reduced the growth of larvae, indicating that interference with *SfV-ATPase* may affect the normal feeding of larvae, while the growth of larvae fed with CHS-dsEGFP showed no difference with control (Table 2). The target gene *SfV-ATPase* of *S. frugiperda* larvae was detected by qPCR for 24 h after feeding LIP-dsRNA complex feed, and it was found that the target gene mRNA level of LIP-dsV-ATPase larvae was significantly reduced (Supplementary Figure S4). These results further prove that liposome-encapsulated dsRNA can improve the efficiency of RNAi.

**TABLE 2:** Effects of liposome-encapsulated dsRNA on the growth of *S. frugiperda* larvae.

NPs-dsRNA	Mortality rate (%)	Weight increase (mg)
W + dsV-ATPase	16.67 ± 5.773503b	22.55 ± 6.50a
LIP + dsV-ATPase	41.67 ± 10.40833a	13.80 ± 5.63b
CHS + dsV-ATPase	18.34 ± 7.637626b	17.51 ± 4.54ab

Note: *V-ATPase*, an important gene for the growth and development of *S. frugiperda* (Parsons et al., 2018). dsV-ATPase is combined with two nanomaterials, liposome-encapsulated dsRNA (LIP-dsV-ATPase) and chitosan-encapsulated dsRNA (CHS + dsV-ATPase), using water instead of nanomaterials as a control (W+ dsV-ATPase). Apply the same amount of NPs-dsRNA to the artificial feed every day and feed the first instar larvae continuously for 1 week. Count the final mortality and weight gain. Five larvae were a biological replicate, and this experiment was performed three times in total. The data shown are mean ± SE, *n* = 15 for the mortality and weight increase calculation, different letters indicate a significant difference among treatments (*p* < 0.05, one-way ANOVA followed by Duncan’s multiple range test).

**FIGURE 5:**
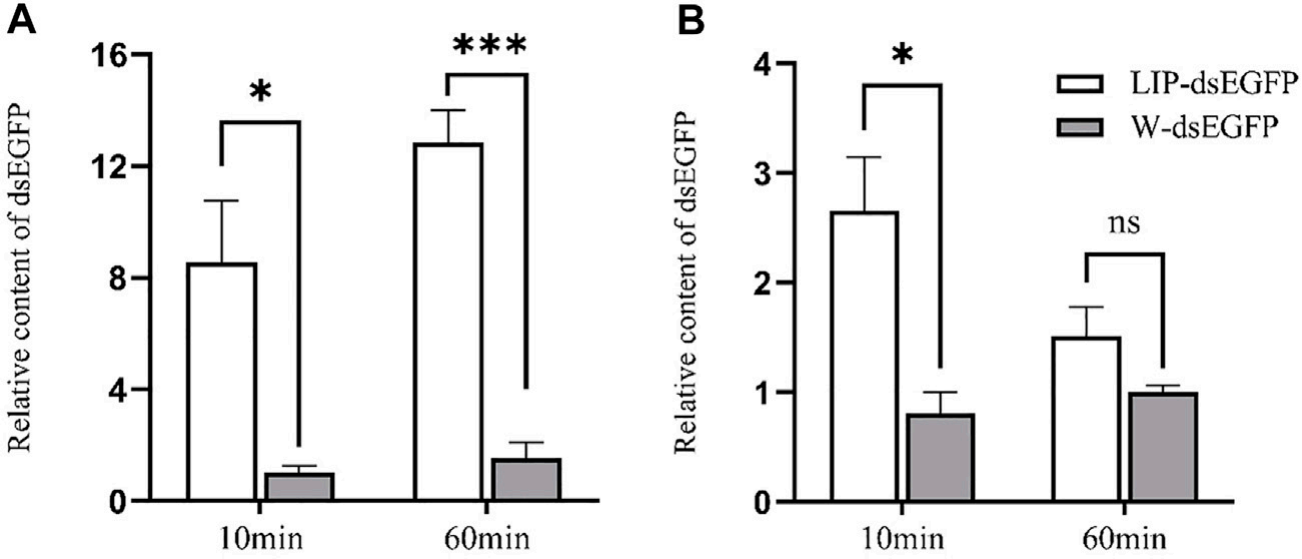
*In vitro* degradation of Lip-dsRNA in midgut juice and hemolymph. The relative content of dsRNA was negatively correlated with the ability to degrade dsRNA. Degradation of liposome-encapsulated dsRNA in midgut juice **(A)**. Degradation of liposome-encapsulated dsRNA in hemolymph **(B)**. Liposome-encapsulated dsRNA (LIP-dsEGFP), dsRNA not encapsulated with liposomes, replaced liposomes with the same amount of water as a control (W-dsEGFP). The two forms of dsRNA were incubated with midgut juice and hemolymph for 10 and 60 min at room temperature, respectively. The dsRNA content after incubation was detected by qPCR, and the relative content ratio was calculated. The data shown are mean ± SE, *n* = 12 for the dsRNA relative content ratio calculation (One-way ANOVA, the least significant difference (LSD) test, ns = No significant difference, *p < 0.05, ***p < 0.001).

To further explore the potential mechanism of enhancing RNAi effect by lipid, the intestinal fluid and hemolymph of the fourth instar larvae were extracted and incubated with LIP-dsRNA *in vitro*. The data shows that in midgut juice, after incubation for 10 and 60 min, the relative content of dsEGFP without liposome encapsulation is significantly lower than that of liposome-encapsulated dsEGFP. After 60 min, the relative content of liposome-encapsulated dsRNA was nearly 12-fold higher than the control due to further degradation of dsEGFP (Figure 5A). In the hemolymph, after 10 min of incubation, the relative content of dsEGFP not encapsulated with liposomes was significantly lower than that of dsEGFP encapsulated with liposomes, but there was no significant difference compared with the control after 60 min (Figure 5B). These results indicate that LIP-dsRNA can improve the stability of dsRNA in the midgut fluid of *S. frugiperda*, thereby increasing the efficiency of RNAi.

### *S. frugiperda* Nuclease Gene is Involved in the Degradation of dsRNA in the Midgut and Hemolymph

**FIGURE 6:**
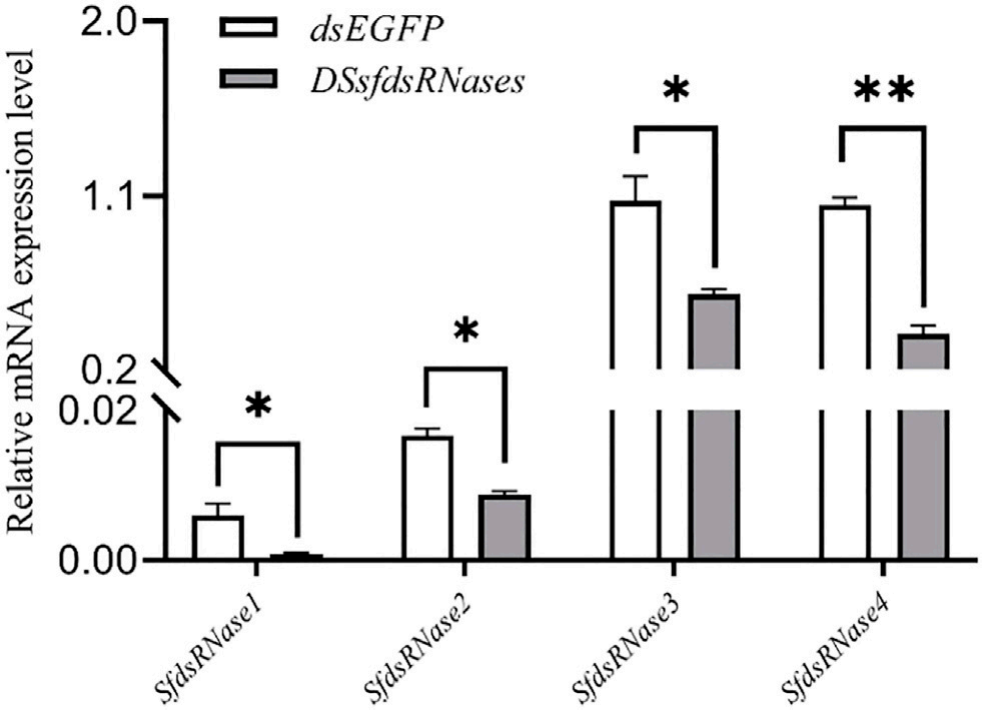
Lip-dsRNA down-regulates the expression of *sfdsRNase*. DssfdsRNase represents the injection of 4 dsRNA of sfdsRNases into the fourth instar larvae, and the control injection of the same amount of dsEGFP. After 24 h, the expression level of sfdsRNase is measured by RT-qPCR. The data shown are mean ± SE, *n* = 12 for the Relative mRNA expression measurement of *sfdsRNases* （One-way ANOVA, the least significant difference (LSD) test, *p < 0.05, **p < 0.01）.

To verify the function of the *sfdsRNase1–4* genes of *S. frugiperda*, we synthesized dsRNA targeted to those four *sfdsRNases* and used the efficient RNAi delivery method described above. To achieve a more obvious RNAi effect, dsRNAs with lip-encapsulated were injected into fourth instar larvae and significant depression phenomena were observed after 24 h. The results showed that the expression of the four *sfdsRNase* genes was significantly downregulated respectively, compared to the control (Figure 6).

**FIGURE 7:**
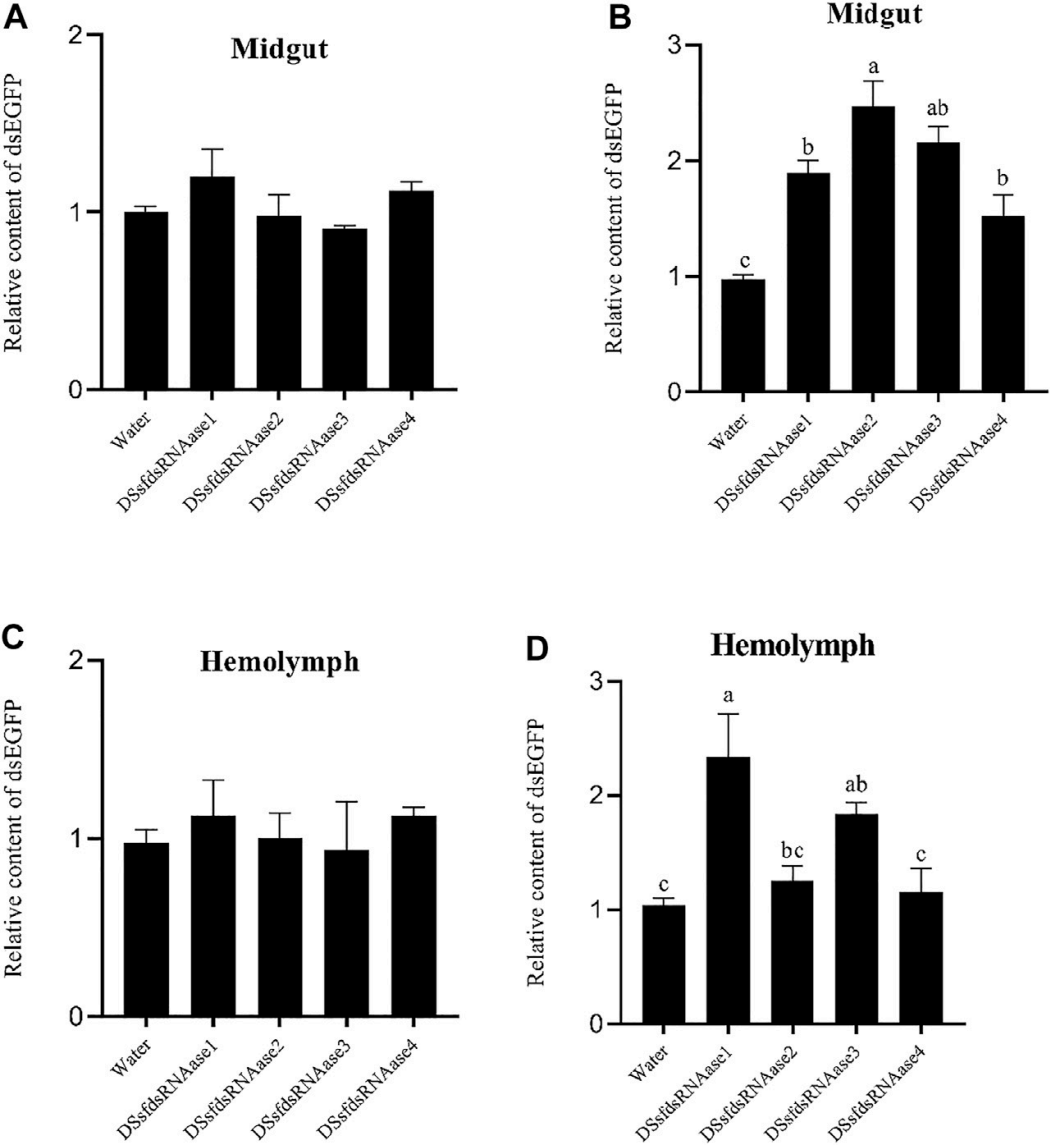
*In vitro* incubation of dsRNA and midgut juice and hemolymph. Relative content of dsEGFP in the control group = 1. The relative content of dsRNA was negatively correlated with the ability to degrade dsRNA. *In vitro* incubation of dsRNA and midgut fluid, dsRNases are the midgut extract of *S. frugiperda* larvae after successfully interfering with 4 *sfdsRNase* genes. At the same time, to avoid the influence of dsEGFP injection on the detection of dsEGFP content after incubation, the larval midgut fluid injected with water was used as a control. The midgut extract was incubated with dsEGFP for 10 and 60 min at room temperature, and the midgut juice was replaced by water as a control. The dsRNA content after incubation was detected by qPCR, and the relative content ratio was calculated [**(A)** represented relative content of dsEGFP after 10 min in midgut, **(B)** represented relative content of dsEGFP after 60 min in midgut, **(C)** represented relative content of dsEGFP after 10 min in hemolymph, **(D)** represented relative content of dsEGFP after 60 min in hemolymph**]**. The data shown are mean ± SE, *n* = 12 for the dsRNA relative content ratio calculation, different letters indicate a significant difference among treatments [*p* < 0.05, one-way ANOVA followed by Duncan’s multiple range test for **(A,B)**].

Next, the midgut juice and hemolymph were dissected from those successful silencing insects and incubated with dsEGFP for 10 and 60 min at room temperature, respectively. The results showed that in the midgut juice, there was no significant difference between all treatment groups and the control at 10 min (Figure 7A). After 60 min, while the relative content of dsEGFP was significantly higher than that in the control group especially after silencing with *sfdsRNase2* and *sfdsRNases3*，and the relative content of silencing *sfdsRNase2* was 2.5 times that of the water control, indicating all the *sfdsRNases* participated in exogenous dsRNA degradation (Figure 7B). A similar situation was detected in hemolymph, there was no significant difference between the treatment group and the control after 10 min incubation (Figure 7C). However, we found the ratio of dsEGFP from *sfdsRNase1* and *sfdsRNase3* treatment groups was much higher than that in other groups after 60 min incubation. Among them, the relative content of the treatment group after silencing *sfdsRNase1* was nearly twice that of the control (Figure 7D). The results suggest that *sfdsRNase1* and *sfdsRNase3* may be involved in the degradation of dsRNA in the hemolymph.

## Discussion

The development of RNAi encloses great potential to be strategies for pest control management (Baum et al., 2007; Song et al., 2019; Kunte et al., 2020; Zhu and Palli, 2020). However, the insensitive RNAi in different insects hindered the application of RNAi in practical fields. As one of the most important insects, the lepidopteran insect caused severe damages to crop. Unfortunately, those insects showed low efficiency to RNAi compared to coleopteran insects, including fall armyworm *S. frugiperda* (Christiaens et al., 2020). As noted, fast dsRNA degradation is one of the key factors affecting RNAi efficiency (Song et al., 2017; Spit et al., 2017). The dsRNases were well documented to play crucial roles in degrading dsRNA, study of *dsRNase* gene family would improve the understanding of RNAi mechanism and make contributions to better usage of RNAi.

To identify potential roles of dsRNases in reduced RNAi efficiency, a genome-scale search was carried in the presented study, and four homolog *dsRNase* genes were identified from *S. frugiperda* genome, named as *SfdsRNase1–4* based on their homology to *S. litura* (Peng et al., 2020). The endounuclease_NS domain was predicted from each protein (Table 1; Supplementary Figure S2), indicating they belong to DNA/RNA non-specific endonuclease family. In addition, those genes were clustered with other lepidopteran insect *dsRNases* from phylogenetic tree analysis, which also supported they are indeed *dsRNase* homolog (Figure 2). However, the number of dsRNases from each lepidopteran and hemipteran (insensitive to RNAi) varies from three to six, while most Coleopteran insects shared one to four homologs (Figure 2; Supplementary Table S1). Lepidopteran insects have evolved multiple copies of *dsRNase* homologs than other insects, therefore those expansions of *dsRNase* genes would be part of an explanation of low RNAi efficiency in lepidopteran and hemipteran insects. A detailed functional analysis of a specific gene is needed to be revealed. Furthermore, those functional hypotheses were supported by the expression profile (Figures 3, 4). General, most dsRNases were highly expressed in midgut and elder larvae, indicating an increased activity, and similar results were found in *S. exigua* and *S. litura*. They found a positive correlation between lower *dsRNase* activity and lower expression levels in young larvae (Vatanparast and Kim, 2017; Peng et al., 2020).

It is well known that naked dsRNA is vulnerable to degrade in fluid, whereas encapsulating dsRNA would protect it from degradation. To increase the RNAi effect and achieve the RNAi phenotype, three nanomaterials were used *in vivo*, as previously reported (Wang et al., 2020). To date, most related works were conducted in sf9 cells, and satisfying results were obtained in *S. frugiperda* (Gurusamy et al., 2020a; Gurusamy et al., 2020b). In the presence of our data, we found liposome significantly increased RNAi effect with successful target gene from previous work (Lin et al., 2017; Gurusamy et al., 2020b). Similar results were observed from the sf9 cell line and tephritid fruit flies, strong RNAi phenotypes were achieved by complexing dsRNA with liposomes (Tayler et al., 2019; Gurusamy et al., 2020b). In lepidopteran insects, the rapid degradation of dsRNA in the midgut and hemolymph is an important factor that limits the efficiency of RNAi. Therefore, to verify whether LIP-dsRNA can enhance the stability of dsRNA in the midgut and hemolymph, thereby improving the efficiency of RNAi. We detected dsRNA content from different time points after incubation with insect midgut and hemolymph and found those dsRNAs were more persistent than the naked ones (Figure 5). Together with the results from *in vivo* and *ex vivo* cell experiments, we speculated the liposome was mainly involved in protecting dsRNA from degradation by endonucleases and improved delivery of intact dsRNA into cells. Studies had also confirmed that oral administration of liposome-encapsulated dsRNA (*α-tubulin*) to German cockroaches *Blattella germanica* can reduce the degradation of dsRNA in the midgut and increase mortality (Lin et al., 2017). Furthermore, we used this method to conduct RNAi experiment targeted with different *SfdsRNase*, a desirable effect was observed (Figure 7). Therefore, the presence of our work provided successful examples for further RNAi experiments in *S. frugiperda*. While the CQD showed the lethal effect on *S. frugiperda* larvae, this result is quite different from works in striped stem borer (*Chilo suppressalis*) (Wang et al., 2020). However, we used the same dosage of CQD as reported in Wang’s work, and we did not decrease the concentration of CQD and tested the lethal effect. Therefore, we deem this situation as the variation of sensitiveness to CQD among different insects.

The role of dsRNase has been demonstrated by several insects and is mainly involved in the degradation of dsRNA in the midgut and hemolymph (Wang et al., 2016; Song et al., 2017; Prentice et al., 2019; Song et al., 2019). Therefore, the variation of insect RNAi efficiency may be the consequence of fast degrading exogenous dsRNA by endogenous dsRNases. The RNAi effect could be induced by considerable dose of dsRNA and most RNAi response is dependent on the concentration of dsRNA (Scott et al., 2013; Wang et al., 2015; Killiny and Kishk, 2017; Peng et al., 2020). In addition, most successful insect RNAi cases were reported from injection method than ingestion due to rapid dsRNA degradation before entering gut epithelial cells (Luo et al., 2013; Sapountzis et al., 2014; Joga et al., 2016; Wang et al., 2016). Therefore, impairing endogenous nucleases and protecting dsRNA from dsRNases degradation would be a feasible solution to improve insect RNAi efficiency. Consequently, a clear understanding of specific nuclease which is involved in dsRNA degradation is urgently needed. The *ex vivo* assays (incubation dsRNA with midgut juice or hemolymph) demonstrated depression of any of sfdsRNases increased the dsRNA persistence in midgut fluid (Figures 7A,B). In addition, silencing of two dsRNases (*sfdsRNase1* and *sfdsRNase3*) also reduced the ability of dsRNA degrading in hemolymph (Figures 7C,D). In *S. litura*, CRISPR-Cas9 mediated knockout of *SldsRNase1* and *SldsRNase2* increased dsRNA stability both in the gut and hemolymph (Peng et al., 2021). In the research of sweetpotato weevil *Cylas puncticollis*, they also found dsRNA degradation in the samples injected with dsCp-dsRNase-3 was significantly reduced (Prentice et al., 2019). Powell et al. (2017) demonstrated a clear relationship between nuclease activity and RNAi effect *in vivo*. Silencing of digestive secretions expressed nuclease enhancing orally delivered RNAi effect in the small hive beetle *Aethina tumida*, which is insensitive to oral RNAi. Hence we believe our experiment provided a feasible scheme for the functional identification of dsRNase in insects. In the presence of our data, we found *sfdsRNase3* participated in both midgut and hemolymph dsRNA degradation. Therefore, we believe *sfdsRNase3* plays an important role in RNAi degradation of *S*. *frugiperda* larvae, and further work needs to be done to illustrate the process and mechanism of potential roles of *sfdsRNase3* affecting dsRNA stability *in vivo* and make practical use of the RNAi effect after the depression of *sfdsRNase3*. Successful application of RNAi by oral-delivery dsRNA is challengeable but overcoming these challenges will be greatly paid back in the field of pest control.

In summary, the characterized *dsRNases* genes from *S*. *frugiperda* were consistent with other reports from other insects, those dsRNases would contribute to dsRNA instability and lead to RNAi recalcitrance as other lepidopteran insects. Furthermore, analysis of dsRNases gene characters and their function from other insects would be helpful to make a solid conclusion whether the existence of multiple dsRNases is a general phenomenon and contribute to low RNAi efficiency in all lepidopteran insects. In addition, we found transfection reagents liposomes greatly protect dsRNA from nuclease degradation in *S*. *frugiperda*. Therefore, co-delivery of nuclease- and specific-dsRNA by Lip-encapsulated dsRNA is a good strategy for enhancing dsRNA stability in *S*. *frugiperda*, and hopefully improving RNAi efficiency. Hence RNAi-mediated pest control approach can be utilized in more practical applications. However, most dsRNase shared high similarities, and it is worth knowing whether the off-target effect was existence after suppression of any dsRNases. In addition, whether inhibition of cellular nucleases would affect the core RNAi mechanism is another critical issue that need to be clarified.

## Data Availability

The original contributions presented in the study are included in the article/Supplementary Material, further inquiries can be directed to the corresponding authors.
