# Supplementary material for: Multiple dsRNases Involved in Exogenous dsRNA Degradation of Fall Armyworm Spodoptera frugiperda
Source: Front Physiol. 2022 May 5;13:850022. doi: 10.3389/fphys.2022.850022 (PMC9117646; doi:10.3389/fphys.2022.850022)

| Species | Organisms | Gene name | Numbers | GenBank accession |
| --- | --- | --- | --- | --- |
| Lepidoptera | *Spodoptera frugiperda* | *S. frugiperda-1* | 4 | OL960003 |
|  |  | *S. frugiperda-2* |  | OL960002 |
|  |  | *S. frugiperda-3* |  | OL960004 |
|  |  | *S. frugiperda-4* |  | OM001111 |
|  | *Spodoptera litura* | *S. litura-1* | 5 | QJD55608.1 |
|  |  | *S. litura-2* |  | QJD55609.1 |
|  |  | *S. litura-3* |  | QJD55610.1 |
|  |  | *S. litura-4* |  | QJD55611.1 |
|  |  | *S. litura-5* |  | QJD55612.1 |
|  | *Bombyx mori* | *B. mori-1* | 3 | NP_001091744.1 |
|  |  | *B. mori-2* |  | XP_012545007.1 |
|  |  | *B. mori-3* |  | XP_004922835.1 |
|  | *Chilo suppressalis* | *C. suppressalis-1* | 4 | AKB95583.1 |
|  |  | *C. suppressalis-2* |  | AKB95584.1 |
|  |  | *C. suppressalis-3* |  | AKB95586.1 |
|  |  | *C.suppressalis-4* |  | AKB95587.1 |
|  | *Danaus plexippus* | *D. plexippus-1* | 3 | EHJ64029.1 |
|  |  | *D. plexippus-2* |  | EHJ63979.1 |
|  |  | *D. plexippus-3* |  | EHJ75678.1 |
| Diptera | *Anopheles darling* | *A. darlingi-1* | 3 | ETN61459.1 |
|  |  | *A. darlingi-2* |  | ETN61460.1 |
|  |  | *A. darlingi-3* |  | ETN62076.1 |
|  | *Culex quinquefasciatus* | *C. quinquefasciatus-1* | 2 | XP_001844830.1 |
|  |  | *C. quinquefasciatus-2* |  | XP_001858177.1 |
|  | *Drosophila melanogaster* | *D. melanogaster-1* | 2 | NP_648610.1 |
|  |  | *D. melanogaster-2* |  | NP_649076.1 |
| Hemiptera | *Acyrthosiphon pisum* | *A. pisum* | 1 | XP_003242653.1 |
|  | *Cinara cedri* | *C. cedri* | 1 | VVC35734.1 |
|  | *Myzus persicae* | *M.persicae* | 1 | XP_022183034.1 |
| Coleoptera | *Tribolium castaneum* | *T. castaneum-1* | 3 | XP_970494.1 |
|  |  | *T. castaneum-2* |  | XP_973011.2 |
|  |  | *T. castaneum-3* |  | XP_008190292.1 |
|  | *Leptinotarsa decemlineata* | *L. decemlineata-1* | 2 | APF31792.1 |
|  |  | *L. decemlineata-2* |  | APF31793.1 |
| Orthoptera | *Schistocerca gregaria* | *S. gregaria-1* | 4 | AHN55088.1 |
|  |  | *S. gregaria-2* |  | AHN55088.1 |
|  |  | *S. gregaria-3* |  | AHN55090.1 |
|  |  | *S. gregaria-4* |  | AHN55091.1 |
|  | *Locusta migratoria* | *L. migratoria-1* | 2 | APF31794.1 |
|  |  | *L. migratoria-2* |  | ARW74135.1 |
|  |  | *L. migratoria-3* |  | ARW74136.1 |

**Table.S1.** Different species dsRNase GenBank accession numbers

**Table.S2**. All primer sequences used in this study

| Primer | Gene name | Forward primer (5’-3’) | Reverse primer (5’-3’) |
| --- | --- | --- | --- |
| Amplification | *sfdsRNase1* | ATGTCTGGCGAATACAGACG | CATCCGACCTCCTTTGTCAG |
|  | *sfdsRNase2* | GCTGTACATAGTCAGGCCCC | ACGTTCACGTTAAAACACAATCG |
|  | *sfdsRNase3* | ATGCGTGCAGTGTTAGTGCT | TTAAGCAAGGAGTCCATTGGTAG |
|  | *sfdsRNase4* | CGAGGCTAGAGCCACTAACG | ACCACGGTGTTATAGCCAGC |
| RT-qPCR | *qsfRNase1* | CGGATTGAAGAGGCCACAGT | CTGAGGAGCAGTGTTGACGT |
|  | *qsfRNase2* | TAGAGGTCAAAAGGCGGCTG | AATGGAAGGTCGCACGTTCT |
|  | *qsfRNase3* | AACAAGTTGTCGTCGCTTGC | AGCATGTGAGCTGACCGAAA |
|  | *qsfRNase4* | CACATCCAACTCACGAGGCT | AACTTCGAGGCGACGTTCAT |
|  | *Sf-βactin* | CGGTATCGTGCTGGACTCCGGTG | GAGTAACCCCTCTCGGTGAGGATC |
| T7-dsRNA | *DSsfdsRNase1* | TAATACGACTCACTATAGGGCAAGGACGTCCGCTGTAACT | TAATACGACTCACTATAGGGTTCGGCCCTAGTATTGCGTC |
|  | *DSsfdsRNase2* | TAATACGACTCACTATAGGGCGGACAGAACGACTGTGCTA | TAATACGACTCACTATAGGGAGCGCAGTTCACGTAATGGA |
|  | *DSsfdsRNase3* | TAATACGACTCACTATAGGGGCTTCGACCAAAACCGTCTG | TAATACGACTCACTATAGGGATACGAGCGCGGAGATTCTG |
|  | *DSsfdsRNase4* | TAATACGACTCACTATAGGGCGAGGCTAGAGCCACTAACG | TAATACGACTCACTATAGGGCGAGCTAAGTATTGCCGGGT |
|  | *dsEGFP* | TAATACGACTCACTATAGGGAAGTTCAGCGTGTCCG | TAATACGACTCACTATAGGGCACCTTGATGCCGTTC |
|  | *dssfV-ATPase* | TAATACGACTCACTATAGGGGAGGCTCTTCGTGAGATCTCAGG | TAATACGACTCACTATAGGGGAAACGATCGTATGACGAGTAGCTG |

**Figure Legend**

**Fig.S1**. Standard curve of dsEGFP. Different dsEGFP content corresponds to different CT values, log10 represents the dsEGFP content in each 20μl sample, and the linear regression model is used to establish the equation.

**Fig.S2.** Schematic diagram of the conserved domain of *S. frugiperda* dsRNase.

**Fig.S3.** The mortality of nanomaterials to *S. frugiperda* larvae. Three candidate nanomaterials were incubated and combined with dsEGFP to form nano-dsRNA solutions (CQD-dsEGFP, LIP-dsEGFP, CHS-dsEGFP), and water was used instead of nanomaterials as a control (W-dsEGFP). Several kinds of dsRNA were smeared on the artificial feed, and the first instar larvae were fed continuously for a week, and finally the mortality of different treatments was calculated. The data shown are mean ± SE, n =12 ,different letters indicate a significant difference among treatments (P < 0.05, one-way ANOVA followed by Duncan’s multiple range test for a，b).

**Fig.S4.** LIP-dsRNA causes significant down-regulation of target gene expression. RT-qPCR analysis was performed on the target gene *SfV-ATPase* of larvae fed LIP-dsRNA feed for one week, and the larvae fed LIP-dsEGFP were used as control .The data shown are mean ± SE, n = 6 for the *SfV-ATPase* relative expression,（One-way ANOVA, the least significant difference (LSD) test, *P < 0.05.）


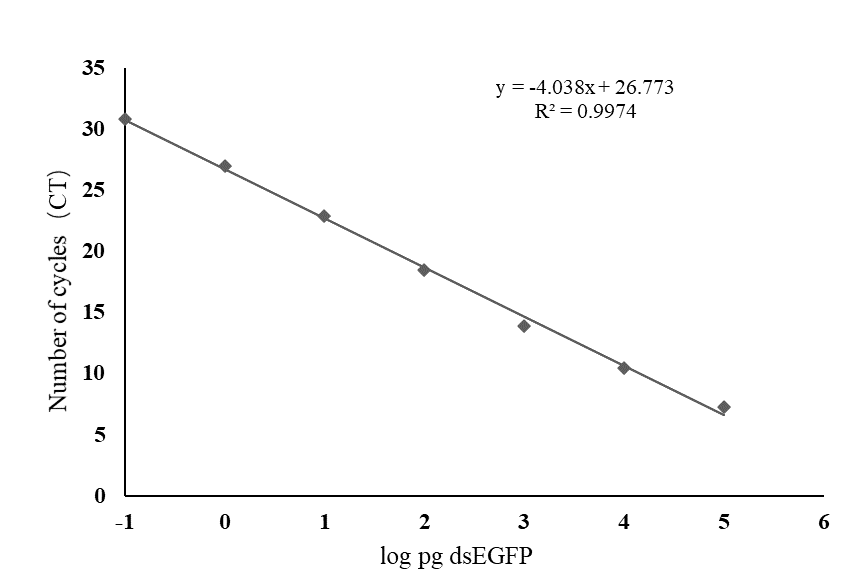


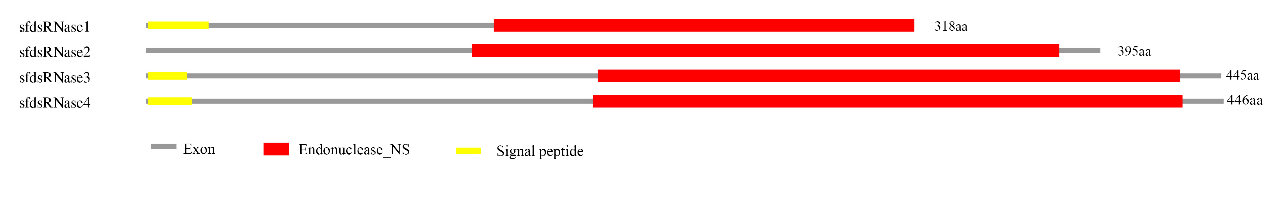


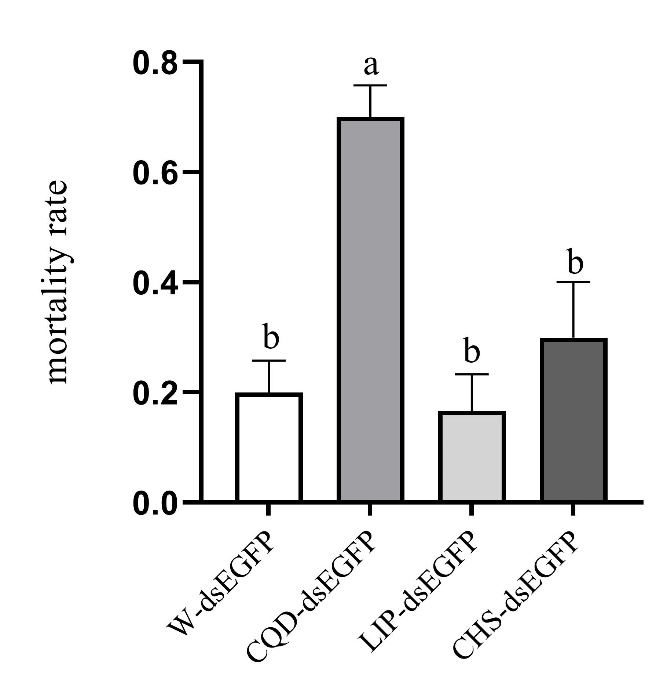


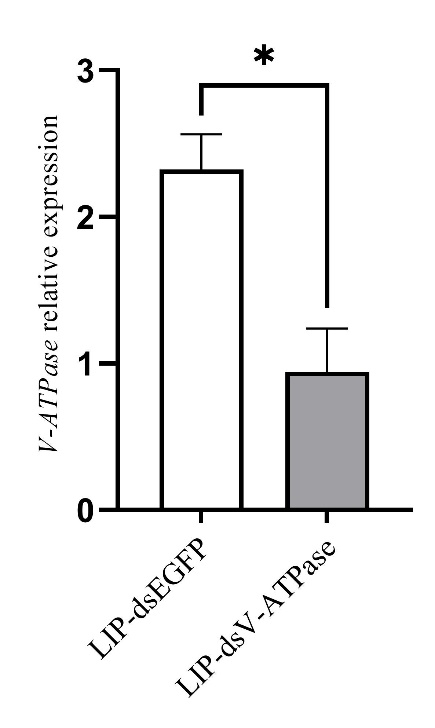

Supplement: Supplementary file 1 [file DataSheet1.docx]
